# Geometric characterization of an electromagnetic surface tracking system in a radiation therapy environment

**DOI:** 10.1002/acm2.70187

**Published:** 2025-07-15

**Authors:** Mumtaz Hussain Soomro, Junliang Xu, Jie Ding, Jochen Cammin, Narottam Lamichhane, Alex Van Slyke, Xiao Liang, Steve Roys, Jiachen Zhuo, Thomas Ernst, Rao P Gullapalli, Erez Nevo, Amit Sawant

**Affiliations:** ^1^ Department of Radiation Oncology University of Maryland School of Medicine Baltimore Maryland USA; ^2^ Department of Radiation Oncology Yale New Haven Hospital Trumbull Connecticut USA; ^3^ Diagnostic Radiology and Nuclear Medicine University of Maryland School of Medicine Baltimore Maryland USA; ^4^ Robin Medical Inc. Baltimore Maryland USA

**Keywords:** 4D imaging, electromagnetic (EM) tracking, Linac, real‐time tracking, volumetric motion models

## Abstract

**Background:**

Despite advances in image‐guided radiation therapy (IGRT), real‐time, soft‐tissue‐based, volumetric motion monitoring remains unsolved. Integrated MRI+Linac systems are a solution, but are costly and complex. X‐ray and optical photogrammetry‐based systems have their limitations. Surrogate‐based motion models, which use external signals to estimate internal motion, offer an alternative. We explore the feasibility of an electromagnetic (EM) fiducial‐based device integrated with a surrogate‐based motion model for real‐time in‐room volumetric motion monitoring.

**Purpose:**

To assess the feasibility of an EM‐tracking system in the linac room, with an eventual goal of integrating it into an MRI‐compatible system for real‐time volumetric motion monitoring.

**Methods:**

We empirically assessed the impact of gantry rotation and the radiation beam on EM‐tracking accuracy using a sinusoidal motion trajectory (2 cm peak‐to‐peak, 5 s per cycle) programmed into a 2D motion platform. Four EM‐tracking sensors were affixed to the platform, and their recorded trajectories were compared to the programmed motion under various conditions, including static and dynamic gantry positions, with and without radiation beams, and during CBCT acquisition.

**Results:**

The EM‐tracking system faithfully reproduced the programmed sinusoidal motion during treatment beam (MV) and CBCT acquisition (kV + gantry rotation). With the beam off and static gantry and static motion platform at 0°, the average point‐wise tracking difference was < 0.5 mm compared to gantry angles of 90°, 180°, and 270°. Similarly, with a moving platform, the sensors achieved a < 1 mm difference at the same angles. Additionally, the gantry's clockwise and anticlockwise rotations caused a < 0.5 mm difference on average at all angles during beam‐off.

**Conclusion:**

Preliminary results show the EM‐tracking system operates with sub‐millimeter accuracy in the linac room, with minimal effects from the radiation beam, gantry motion, or CBCT acquisition, supporting its feasibility for real‐time volumetric motion monitoring during IGRT.

## 1 INTRODUCTION

Respiratory motion induces complex variations in the shape and position of the tumor relative to the radiation beam and adjacent organs at risk (OARs) during thoracic radiotherapy (RT).^1^ These variations can lead to notable geometric and dosimetric inaccuracies, potentially resulting in reduced tumor control and heightened radiation‐induced toxicity.^2^, ^3^, ^4^ Hence, effective motion management strategies are essential in thoracic RT.

Recently, a range of motion management techniques has been explored. These techniques for managing motion during thoracic RT rely primarily on pre‐treatment respiratory‐correlated four‐dimensional computed tomography (4DCT), which captures CT projections over multiple respiratory cycles and sorts them into six to ten volumes representing an average single cycle.^5^, ^6^ Studies have reported tumor motion amplitudes ranging from 0.5 to 2.5 cm, with discrepancies up to 3 mm between phase‐ and amplitude‐based sorting approaches.^5^, ^6^ In parallel, single‐point real‐time monitoring methods such as external surrogates, robotic tracking, and electromagnetic systems have been used to estimate internal tumor motion.^7^, ^8^, ^9^, ^10^, ^11^, ^12^ These have shown that tumor position can vary by more than 5 mm in over 20% of treatment fractions,^7^ and that 4DCT or CBCT can underestimate motion by 3–5 mm.^12^ Electromagnetic tracking has captured motion excursions up to 30 mm with submillimeter precision.^11^ However, these methods often fail to fully capture the complexity of respiratory motion, including variations from cycle to cycle, day‐to‐day breathing changes, and intra‐fraction baseline shifts.^13^, ^14^, ^15^ Later studies have specifically highlighted these limitations: for example, Steiner et al.^14^ found that both 4DCT and 4D‐CBCT under‐predicted lung tumor motion, while Shah et al.^13^ observed intrafraction shifts greater than 5 mm in 30% of treatment sessions. Additionally, modeling approaches using surface motion and volumetric correlation have revealed residual discrepancies of up to 4 mm when relying solely on conventional 4DCT or external surrogates.^15^ These findings suggest that more adaptive or comprehensive motion characterization strategies may be needed in clinical practice.

As thoracic RT advances with techniques such as stereotactic body radiotherapy (SBRT), proton therapy, and particle therapy, it has become increasingly clear that monitoring the tumor target with a single‐point or average breathing cycle is insufficient.^15^ With these more advanced modalities, a single‐point representation or average breathing cycle does not adequately capture the complex motion and interactions within the irradiated volume. Real‐time, soft‐tissue‐based volumetric monitoring is essential to account for the detailed spatiotemporal dynamics, including baseline shifts, translation, deformation, rotation, and interactions between the tumor and surrounding OARs. To ensure precise dose delivery, both high spatial and temporal resolution are necessary before and during treatment.^15^

To address these challenges, volumetric surrogate‐based motion models (SMMs) have been investigated by multiple groups.^1^ These models establish a relationship between internal motion and measurable surrogates, developing a correspondence model based on observed connections between internal anatomical motion and surrogate data. The accuracy of these models depends on the selection of the surrogate, the quality of anatomical motion captured for training, the associated model, and the variability of respiratory motion.^15^ Early volumetric motion models, like those by Blackall et al.^16^ and McClelland et al.,^17^ used affine and deformable registration techniques to model entire volumes of interest. Blackall et al.^16^ used diaphragm motion from fast MRI as a surrogate, differentiating between inhalation and exhalation to capture intra‐cycle variations. This study used dynamic MRI to assess respiratory motion variability and develop motion models for RT planning. They found that diaphragm motion varied by up to 20 mm intra‐cycle and that inter‐cycle variability could exceed 5 mm, emphasizing the limitations of single‐cycle models. The study also noted differences of up to 12 mm in diaphragm position between inhalation and exhalation across cycles, underscoring the need for multi‐cycle modeling. At the same time, McClelland et al.^17^ utilized anterior‐posterior chest displacement from 4DCT to address inter‐cycle variations. They developed a continuous 4D motion model from multiple respiratory cycles using 4DCT and deformable image registration. They reported that tumor position could vary by up to 8 mm between cycles, even within a short acquisition period. Their method improved modeling accuracy compared to single‐cycle approaches, with residual errors in predicted motion reduced to 1.5–2 mm on average.

Our research group has developed a volumetric motion monitoring method that overcomes several limitations inherent in current practices, such as 4DCT simulation and single‐point real‐time monitoring. This approach involves creating a volumetric motion model based on various respiratory cycles, utilizing 4DCT in conjunction with real‐time surface photogrammetry (optical‐surface monitoring).^15^ The model is updated for each treatment session using in‐room kV fluoroscopy to adjust for day‐to‐day variations between external surface movement and internal anatomy. Additionally, kV fluoroscopy is used during treatment sessions to refine the model for intra‐fractional changes. Clinical testing of this model in a prospective study with lung RT patients demonstrated significantly improved spatiotemporal accuracy compared to traditional 4DCT methods.^15^ However, their optical surface monitoring system has line‐of‐sight limitations that make it unsuitable for an MRI room.

This study focuses on evaluating the feasibility of integrating an electromagnetic fiducial‐based tracking system into the clinical setting for real‐time motion monitoring during imaging‐guided radiotherapy (IGRT). By incorporating electromagnetic tracking, this system addresses a key limitation of surrogate‐based systems, as it does not rely on a line‐of‐sight, thus providing enhanced flexibility in monitoring tumor motion.

The goal of this study is to directly assess the practical integration of the Aurora EM tracking system [Northern Digital Inc. (NDI)] into a clinical RT setting for real‐time volumetric motion tracking. One concern with electromagnetic sensor/receiver‐based tracking is that the presence of massive metallic components in the linac head and their motion may cause interference with the EM signals, resulting in signal loss or erroneous readings. In this work, we characterize the tracking accuracy of this system under a variety of scenarios relevant to modern image‐guided radiation therapy—static and moving motion platform to represent static and moving patient anatomy sites, and with static and rotating gantry to represent CBCT acquisition or volumetric modulated arc treatments, as well as the presence of MV radiation.  The value of this study is to establish the initial feasibility of the EM‐based surface tracking approach under clinical IGRT conditions, which is a critical step before comprehensive in‐patient characterization.

## 2 MATERIALS AND METHODS

**FIGURE 1:**
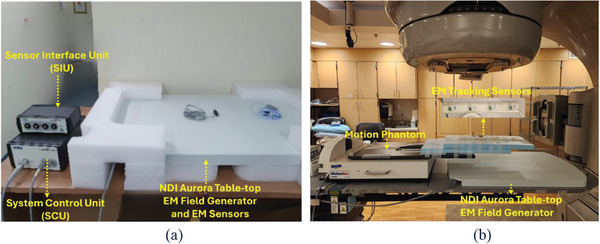
(a) NDI system's components. (b) Experimental setup.

In this study, we aim to assess the feasibility and performance of the NDI Aurora EM tracking system (EMTS) in the linac environment. The components of the EMTS system and the experimental setup are shown in Figure 1. In this section, we will cover three main parts. The first part will focus on the NDI Aurora EMTS and its components. The second part will introduce the 2D motion platform, which is used to assess the stability of the NDI EMTS. The third part will discuss the experiment carried out under various conditions inside the linac room to evaluate the reliability and motion‐tracking ability of the NDI EMTS.

### 2.1 NDI Aurora EM tracking system (EMTS)

The NDI Aurora is an advanced electromagnetic tracking system used in medical applications for accurate real‐time tracking.^18^, ^19^, ^20^ It utilizes electromagnetic fields to determine the position and orientation of sensors in space, providing six degrees of freedom for comprehensive tracking.^20^

The system is designed to function without a direct line of sight, making it highly useful in clinical environments.^20^ Components of the system include the tabletop field generator, EM sensors, sensor interface unit (SIU), and system control unit (SCU), as shown in Figure 1a. These components are made of printed circuit boards (PCBs). The tabletop field generator emits an electromagnetic field while the sensors’ signals enable the EMTS to detect their position and orientation within the field. The SIU aggregates data from multiple sensors and transmits it to the SCU for processing. The SCU calculates the real‐time location of each sensor and integrates it with software applications for real‐time display and analysis.^20^

### 2.2 2D motion platform

To emulate chest wall motion due to breathing, we have used the MotionSimXY motion platform (Sun Nuclear, Melbourne, Florida, USA). A strip containing 4 EM‐tracking sensors was affixed to the 2D motion platform as shown in Figure 1b. The 2D motion platform is programmed with a sinusoidal trajectory (2 cm peak‐to‐peak; 5 s/cycle) and repeated for 60 and 75 s. To characterize the effect of potential signal distortion/degradation due to the linac's gantry motion and/or radiation, we compared the motion trajectories recorded by the EM sensors with those programmed into the motion platform. We used the Python module pyBOAT^21^ to smooth EM sensors’ output by removing unwanted high‐frequency components.

The NDI Aurora EMTS, under ideal conditions, is specified by the manufacturer to have a tracking accuracy of RMS error = 0.48 mm and 95% confidence interval = 0.88 mm^1^, although actual performance may vary depending on the surrounding electromagnetic (EM) environment. Due to the absence of a reliable external gold standard ground truth system, direct validation of the programmed versus actual platform motion was not feasible. The platform may exhibit small deviations from its programmed motion—up to 0.4 mm in amplitude and 4% in frequency—based on internal specifications. Therefore, we adopted an indirect validation approach that leverages the platform's structural rigidity: EM sensors were rigidly affixed to the platform, which does not undergo deformation under the low‐inertia motion used. As a result, the relative distances between the sensors remain constant (up to 30 cm between sensors 1 and 4) throughout the motion cycle. These known geometric distances (measured via ruler) serve as a practical internal reference to evaluate the consistency of the EM tracking system under different experimental conditions.

To evaluate the impact of the motion platform inaccuracy and environmental factors on EM tracking performance, we analyzed the relative distances between sensors that were rigidly attached to the motion platform. Since the platform does not deform and the sensors are fixed in position, these distances should remain constant throughout motion. By taking sensor 1 as a reference, we confirmed consistent distances between sensor 1 and sensor 2 (∼100 mm), sensor 1 and sensor 3 (∼200 mm), and sensor 1 and sensor 4 (∼300 mm). Any variation in measured distance reflects tracking inaccuracies rather than platform deformation. This approach allows us to indirectly assess the EMTS performance under different gantry positions, motion states, and imaging/radiation conditions. While a direct comparison to the programmed trajectory was not possible due to small inherent uncertainties in platform motion, this method provides a reliable internal metric to characterize tracking consistency. Using the fixed distances between sensors as a reference enabled a clearer assessment of tracking accuracy, independent of any motion platform‐related discrepancies.

### 2.3 Experiments

To assess the accuracy and reliability of the EMTS within the treatment room, we aimed to examine potential interference with the output of the EM motion sensors caused by the linac's metallic components and other medical equipment. Using the 2D motion platform, we investigated the system's tracking stability and motion‐tracking capabilities under various conditions outlined in Table 1. These conditions included active radiation, cone‐beam CT (CBCT) imaging procedures, dynamic gantry clockwise and anticlockwise rotation through 360^0^ with and without the radiation beam, as well as static gantry positions. Our primary goal in this analysis was to achieve a motion tracking criterion with an error margin of ± 1 mm, ensuring a considerable accuracy.^4^

**TABLE 1:** Experiments under six different conditions.

Experiment setting	Condition
Without radiation 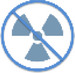	Static Gantry and Static Motion Platform
	Static Gantry and Moving Motion Platform
	Moving Gantry and Static Motion Platform.
	Moving Gantry and Moving Motion Platform.
With radiation 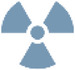	Analyzing the Effect of Radiation (6 MV)
CBCT imaging 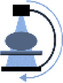	Analyzing the Effect of Cone Beam CT (CBCT) Acquisition (120 kV)

#### 2.3.1 Condition1: Static gantry and static motion platform

For this experiment, we conducted a comparison of the EM sensors’ output from the static motion platform with the linac gantry positioned at 0, 90, 180, and 270 degrees, respectively.

#### 2.3.2 Condition2: Static gantry and moving motion platform

In this condition, the gantry was kept static at 0°, 90°, 180°, and 270°, while the platform was moved along a predefined trajectory for each gantry angle. The objective was to evaluate the reproducibility of the EM sensors’ output at these angles and to compare the output between 0° and 90°, 180°, and 270°.

#### 2.3.3 Condition3: Moving gantry and static motion platform

In this experiment, we rotated the gantry in both clockwise and anti‐clockwise directions while keeping the platform static. The main objective is to analyze the effect of gantry rotation on EM sensors’ output

#### 2.3.4 Condition4: Moving gantry and moving motion platform

In this experiment, we rotated the gantry in both clockwise and anti‐clockwise directions while keeping the platform moving. The main objective is to analyze the effect of gantry rotation on EM sensors’ output when the platform was moving.

#### 2.3.5 Condition5: Analyzing the effect of radiation

In this analysis, we aimed to investigate the impact of radiation beams on the output of EM sensors during both clockwise and anticlockwise gantry rotations. Specifically, we simulated a volumetric‐modulated arc therapy (VMAT) treatment plan, which is commonly used for dynamic, highly conformal treatments.

#### 2.3.6 Condition6: Analyzing the effect of CBCT acquisition

In this case, the CBCT images were obtained by rotating the gantry in both directions to characterize the impact of CBCT acquisition effect on the output of EM sensors and to observe the transition effect when the CBCT acquisition state changes.

## 3 RESULTS

**FIGURE 2:**
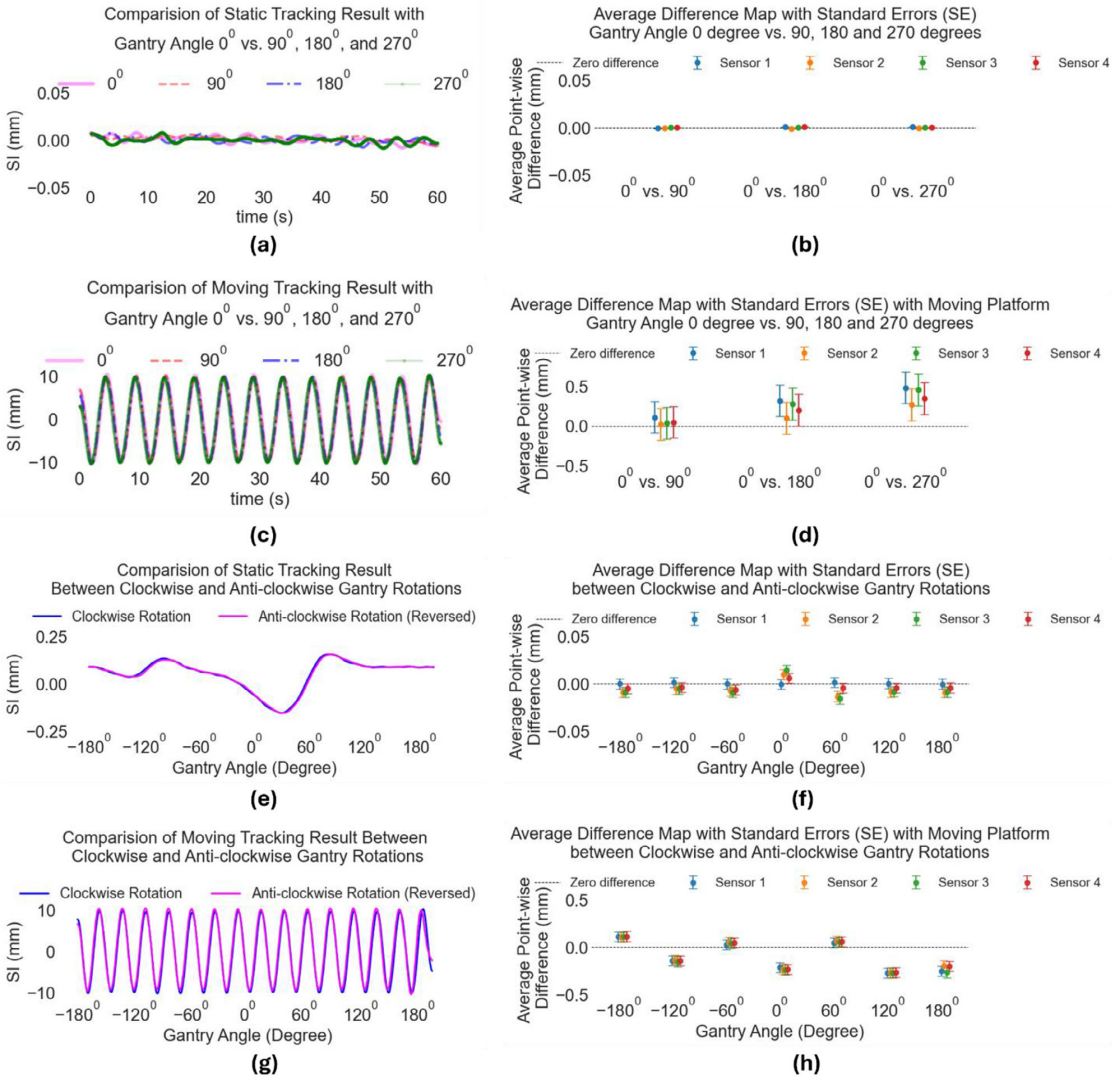
(a,b) Tracked EM sensor motion in Condition 1: comparison of the outputs at gantry angles of 90°, 180°, and 270° with the gantry angle 0° when platform and gantry rotation are static; (c,d) Condition 2: static gantry and moving motion platform; (e,f) Condition 3: comparison between gantry rotation with static motion platform; (g,h) Condition 4: comparison between gantry rotation with moving motion platform. SI is an acronym for superior‐inferior.

Various experimental conditions were evaluated to assess the performance of EM sensors in different gantry rotations and platform motion settings. Under static gantry and static motion platform condition, the outputs at gantry angles of 90°, 180°, and 270° were found to be closely aligned with the output at 0° (Figure 2a), with no significant differences in the point‐wise average (< 0.05 mm) between these angles (Figure 2b). When the gantry remained static and the motion platform was in motion, the EM sensors consistently tracked the motion pattern observed (Figure 2c), with no significant disparities (< 1 mm) observed at the aforementioned angles (Figure 2d). In scenarios where the gantry was rotating in clockwise and anti‐clockwise directions with a static platform, the corresponding EM sensor's output was demonstrated across both rotation directions (Figure 2e), with negligible average point‐wise differences (< 0.05 mm) noted at each angle (Figure 2f). When both the gantry and motion platform were in motion, the EM sensors continued to provide consistent tracking performance across gantry angles (Figure 2g), with minimal variation observed in the readouts (Figure 2h).

**FIGURE 3:**
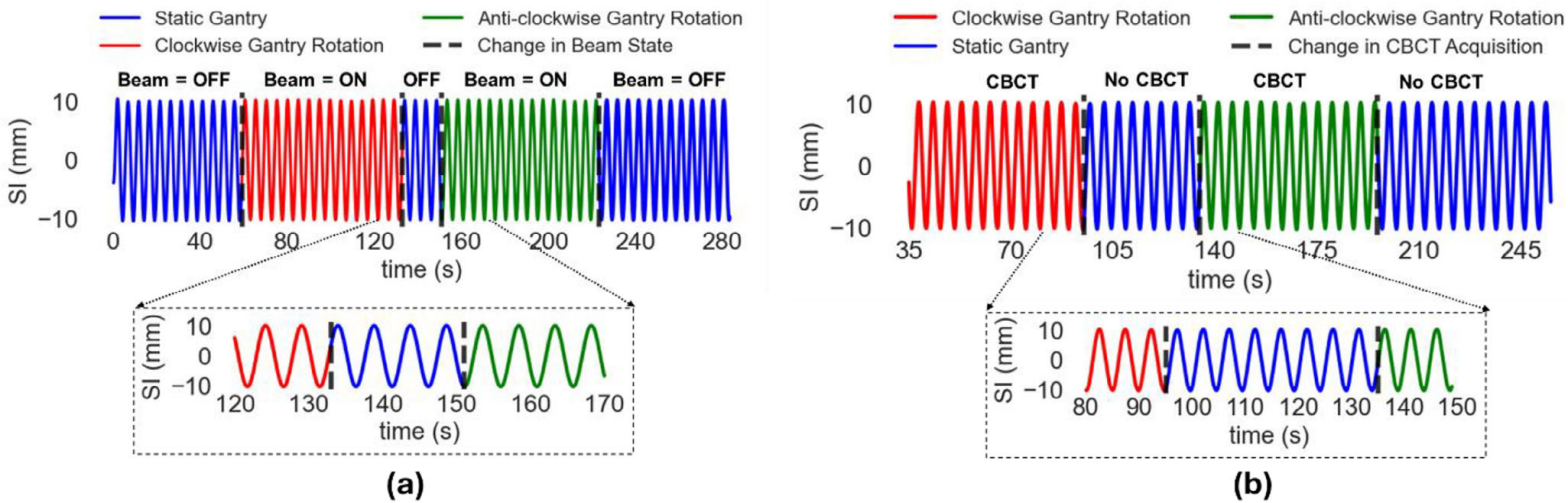
Tracked EM sensor motion for (a) Condition 5: analyzing the effect of radiation; (b) Condition 6: analyzing the effect of cone beam CT (CBCT) acquisition. SI refers to the superior‐inferior direction.

The impact of radiation on sensor performance was also analyzed, and the EM sensors consistently generated output during gantry rotation, with no transitional effects when the radiation beam was turned on or off (Figure 3a). Finally, during cone beam CT (CBCT) acquisition, the EM sensors precisely followed the programmed trajectory in any direction (Figure 3b), with no observable effects when transitioning between CBCT acquisition and non‐acquisition phases.

Figure 2 demonstrates that tracking errors are linked to the movement of the motion platform (compare panels a,b with c,d, and e,f with g,h). We hypothesized that this may be due to inaccuracy in the platform motion itself (which is assumed to be a pure sinusoidal waveform), or due to the adverse effect of the moving platform on the tracking system (e.g., EM interference between the motor of the platform and the tracking system).

Results of this analysis are shown in the supplementary material “Appendix A.” Please see Appendix A—Tables and Figures 1A–4A show high stability of the distance between the sensors, which is similar to tracking results when the motion platform is static, as shown in Figure 2a,b. Please see Appendix A ‐Table and Figures 5A–8A and 11A–12A show fluctuating distances between sensors which indicate that the moving platform adversely affects the tracking system, and this explains the higher errors in Figure 2d and h. The variations in the estimated distance between sensors (please see Appendix A—Tables and Figures 9A and 10A) are similar to the variations in the tracking results of the sensors (Figure 2e,f).

Table 2 tabulates the estimated distances from each experimental condition, indicating minimal variations affirming the sensors' capability to maintain accurate measurements regardless of the motion dynamics introduced by the gantry.

**TABLE 2:** Comparison of distances of sensor 1 vs. 2, 3, and 4, respectively.

Conditions Sensors/Gantry	Distances [mean (mm) ± std(mm)]		
	Sensor 1 vs. 2	Sensor 1 vs. 3	Sensor 1 vs. 4
Condition 1^a^ ^static/static^	101.19 ± 0.07	201.46 ± 0.11	301.35 ± 0.12
Condition 2^a^ ^motion/static^	101.11 ± 0.43	201.38 ± 0.17	301.62 ± 0.35
Condition 3^b^ ^static/motion^	100.56 ± 0.08	201.24 ± 0.11	301.26 ± 0.07
Condition 4^b^ ^motion/motion^	101.12 ± 0.44	201.37 ± 0.18	301.60 ± 0.37
Condition 5 ^radiation^	100.45 ± 0.22	200.18 ± 0.33	N/A
Condition 6 ^cbct^	101.13 ± 0.21	202.00 ± 0.26	N/A

^a^ Average distances of gantry angles 0, 90, 180, and 270 degrees.

^b^ Average distances of gantry clockwise and anticlockwise rotations.

^NA^Sensor 4 was out of order during experiments.

## 4 DISCUSSION AND CONCLUSION

In this study, we explored the feasibility of incorporating an EM surface tracking system into a clinical setting for real‐time monitoring during image‐guided radiotherapy (IGRT). While the integration steps have not been fully implemented in this study, the preliminary test results demonstrate that the system can operate effectively within the clinical environment. Detailed integration processes and clinical implementation will be explored in future work.

Our empirical test results show promising results regarding the system's stability and accuracy, even when dealing with potential interfering factors such as gantry rotation, radiation beams, and CBCT imaging.

The focus of this study was to determine the compatibility of the EM‐tracking system (NDI Inc.) with the operational environment of a linac treatment room. We empirically tested the performance of the EM sensors under various conditions, such as static and dynamic gantry rotations with radiation beam ON and OFF, and during CBCT image acquisition. We found that the EM‐tracking sensors maintained high accuracy, with average differences consistently below 1 mm across all tested scenarios. The system demonstrated stable, real‐time motion tracking performance without significant interference from the linac's metal components, radiation beam, or CBCT imaging. These results highlight the sensors' reliable performance when external platform effects are minimized, supporting their potential for clinical use. While gantry motion does affect tracking accuracy, the impact is minor—approximately sub‐millimeter standard deviation—and may be acceptable for the intended application.

In advancing toward our goal, SMMs play a crucial role. The process of developing these SMMs involves estimating internal lung motion from 4D imaging protocols and correlating it with the concurrently acquired external surrogate to generate new volumetric estimates of internal anatomy for each surrogate observation.^14^, ^15^, ^22^, ^23^, ^24^, ^25^ These models harness either external or internal surrogate signals to forecast the movement of tumors and normal organs within the body. Internal surrogates, like implanted fiducial markers, offer precise tracking but involve invasive procedures. Additionally, fiducial tracking via x‐ray fluoroscopy can expose patients to high skin doses and potential toxicity risks.^26^ Furthermore, x‐ray fluoroscopy has limited contrast when it comes to imaging soft tissues.^26^, ^27^ While MRI can generate high‐quality images of soft tissues,^26^, ^28^ but it is expensive. Most clinics currently only have 2D planar tracking capability in their MRI‐guided RT systems.^26^, ^29^, ^30^ To address these challenges, our research group^15^ and others have explored models utilizing 4D CT and optical imaging techniques, Optical surface imaging systems, such as Vision RT (Vision RT Ltd., London, UK) and Identify (Varian, Siemens Healthineers), have proven effective but are limited by line‐of‐sight constraints, rendering them incompatible with MRI scans. Notably, the Calypso EM system was once a viable option for real‐time target localization and motion management.^31^, ^32^, ^33^ However, the Calypso system is not compatible with MRI, since it may cause artifacts during MRI.^34^ Also, the Calypso EM system is no longer available commercially. The Cyberknife (Accuray) and the Vero (Brainlab) have both offered real‐time motion tracking in clinical settings, which is similar to our proposed real‐time EM motion tracking. Our focus is on providing real‐time volumetric image‐guided motion management, as opposed to single‐point real‐time monitoring. We also recognize the existence of similar systems, such as the Brainlab ExacTrac Dynamic system,^35^ which employs optical and infrared monitoring to correlate CBCT or planar x‐ray images with the body surface at each fraction. Our proposed system, however, offers a non‐line‐of‐sight solution, which could be advantageous in applications such as proton therapy where traditional surface‐guided techniques face limitations. One of the key advantages of the EM tracking system is that it does not rely on a direct line‐of‐sight for motion monitoring. This feature makes it particularly appealing for proton therapy applications, where line‐of‐sight‐based systems, such as SGRT, can be challenging, especially when a range shifter is used with the snout extended. However, evaluating the performance of the EM system in proton therapy will require further testing, which we plan to address in future work.

In addition to the EM tracking system, another promising development in motion monitoring is the integration of MRI+linac systems.^36^, ^37^ Owing to their ability to provide soft‐tissue‐based monitoring, MRI+linacs overcome many of the limitations associated with current in‐room image guidance techniques. Recent advancements in MR+linacs^38^, ^39^ offer superior soft‐tissue contrast and real‐time imaging, providing continuous tumor motion monitoring and adaptive treatment delivery. However, the high cost and operational complexity of these systems limit their accessibility in many clinical settings. The non‐line‐of‐sight advantage of the EM tracking system could offer a cost‐effective alternative to MRgRT, especially for real‐time volumetric motion tracking.

Although this study focused on superior‐inferior (SI) motion using a 2D motion platform, we acknowledge that real respiratory motion includes both anteroposterior (AP) and SI components. Future work will extend this study by utilizing a more flexible lung motion platform to simulate both AP and SI movements. Additionally, while this study primarily used 6MV energy for testing, we recognize the importance of evaluating the system's stability across different beam energies. Future studies will also investigate the system's behavior in different orientations to ensure its robustness and reliability in clinical settings. Additionally, this study primarily utilized 6MV energy for testing. However, we recognize the importance of evaluating the EM tracking system's stability across a range of beam energies (e.g., 6X, 10X, 15X, and FFF) to ensure its robustness and clinical applicability in different treatment settings. Future studies will address the dosimetric properties of the EM tracking system, including its potential interference with patient setup and radiation delivery. The ultimate goal of our research is to use the EM tracking system as a surrogate to develop a motion model that can enhance the accuracy and precision of motion management during RT. Although the integration of the EM system with an SMM is a key goal of our research, it was not demonstrated in this study. We plan to present this integration in future work, where we will describe the process in greater detail.

In conclusion, our study demonstrates that the EM tracking system remains stable in the linac treatment environment, even during treatment delivery and CBCT acquisition, with sub‐mm level accuracy. The results highlight the potential of EM surface tracking as a viable solution for real‐time monitoring of surface motion in RT. Future studies will extend this work to incorporate more complex motion patterns, test additional beam energies, and explore the integration of the EM system with SMMs to improve clinical motion management.

## AUTHOR CONTRIBUTIONS

All authors contributed equally to this work.

## CONFLICT OF INTEREST STATEMENT

The authors declare no conflicts of interest.

## Supporting information



Supporting Information

## Data Availability

The data that support the findings of this study are available from the corresponding author upon reasonable request.

## Footnotes

1 Manufacturer specifications available at: https://www.ndigital.com/electromagnetic‐tracking‐technology/aurora/
