# Supplementary material for: Geometric characterization of an electromagnetic surface tracking system in a radiation therapy environment
Source: J Appl Clin Med Phys. 2025 Jul 15;26(7):e70187. doi: 10.1002/acm2.70187 (PMC12260265; doi:10.1002/acm2.70187)

Appendix A

Comparison of distances for sensor 1 vs. 2, sensor 1 vs. 3, and sensor 1 vs. 4.

Condition1: Static Gantry and Static Motion Platform

Table 1A: Sensors' distances comparison when gantry angle = 0 degrees

Metrics (mm)	Sensor 1 vs. 2	Sensor 1 vs. 3	Sensor 1 vs. 4
**mean (mm)**	101.213633	201.471349	301.401774
**std (mm)**	0.003941	0.003647	0.003983

Figure 1A. Sensors Distances for Static Plateform at Gantry Angle 0^0

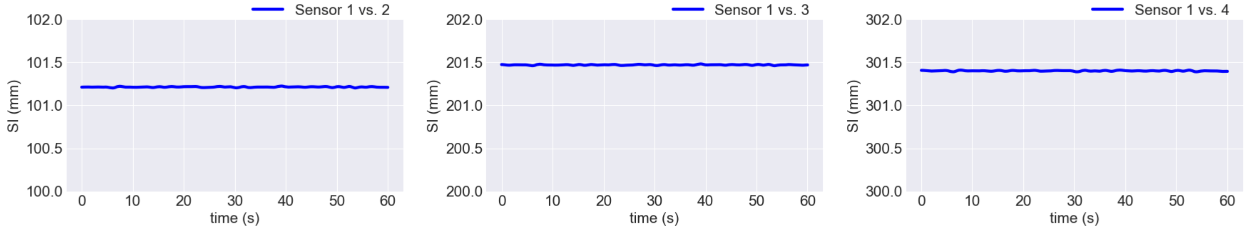

Table 2A: Sensors' distances comparison when gantry angle = 90 degrees

Metrics (mm)	Sensor 1 vs. 2	Sensor 1 vs. 3	Sensor 1 vs. 4
**mean (mm)**	101.076374	201.278116	301.169269
**std (mm)**	0.003366	0.003341	0.003849

Figure 2A. Sensors Distances for Static Plateform at Gantry Angle 90^0

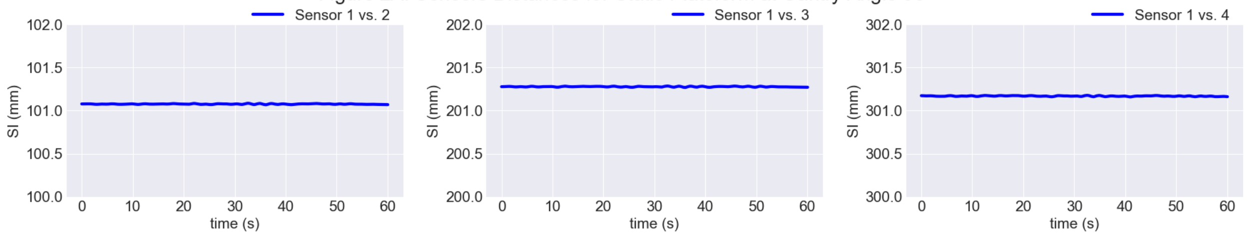

Table 3A: Sensors' distances comparison when gantry angle = 180 degrees

Metrics (mm)	Sensor 1 vs. 2	Sensor 1 vs. 3	Sensor 1 vs. 4
mean (mm)	101.209599	201.497502	301.337372
std (mm)	0.004143	0.004228	0.004098

Figure 3A. Sensors Distances for Static Plateform at Gantry Angle 180^0

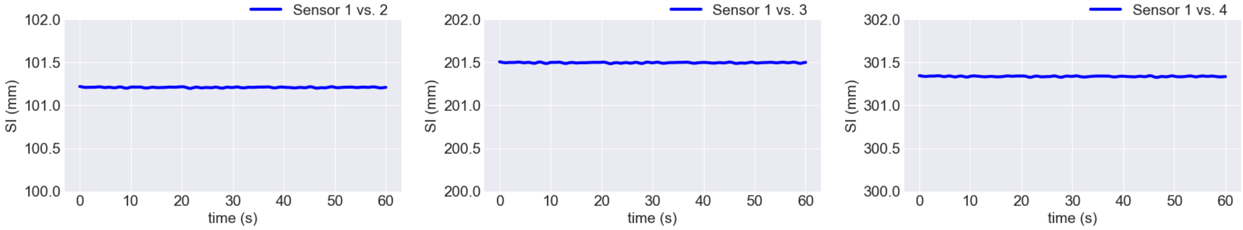

Table 4A: Sensors' distances comparison when gantry angle = 270 degrees

Metrics (mm)	Sensor 1 vs. 2	Sensor 1 vs. 3	Sensor 1 vs. 4
mean (mm)	101.259375	201.589382	301.496541
std (mm)	0.003592	0.003489	0.004084

Figure 4A. Sensors Distances for Static Plateform at Gantry Angle 270^0

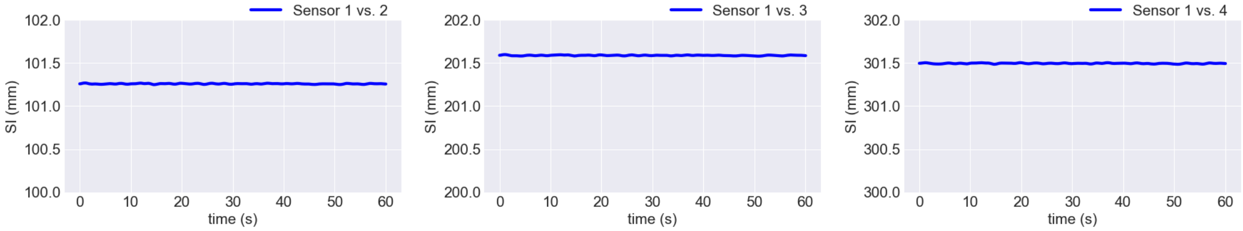

Condition2: Static Gantry and Moving Motion Platform

Table 5A: Sensors' distances comparison when gantry angle = 0 degrees

Metrics (mm)	Sensor 1 vs. 2	Sensor 1 vs. 3	Sensor 1 vs. 4
mean (mm)	101.095750	201.368281	301.599125
std (mm)	0.394334	0.143013	0.294421

Figure 5A. Sensors Distances for Moving Plateform at Gantry Angle 0^0

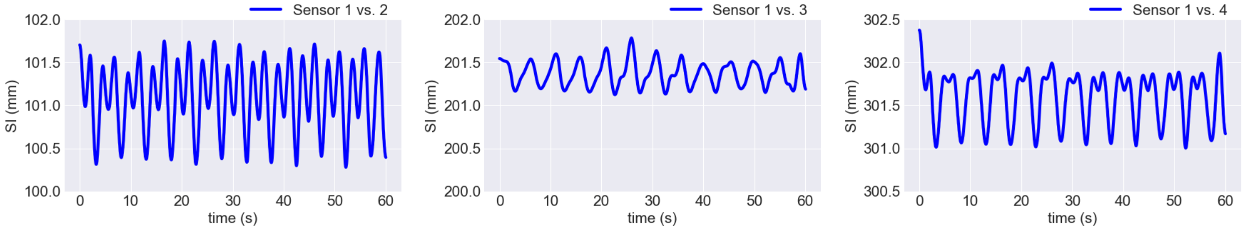

Table 6A: Sensors' distances comparison when gantry angle = 90 degrees

Metrics (mm)	Sensor 1 vs. 2	Sensor 1 vs. 3	Sensor 1 vs. 4
**mean (mm)**	101.006486	201.249261	301.533380
**std (mm)**	0.428125	0.155488	0.349820

Figure 6A. Sensors Distances for Moving Plateform at Gantry Angle 90^{0}

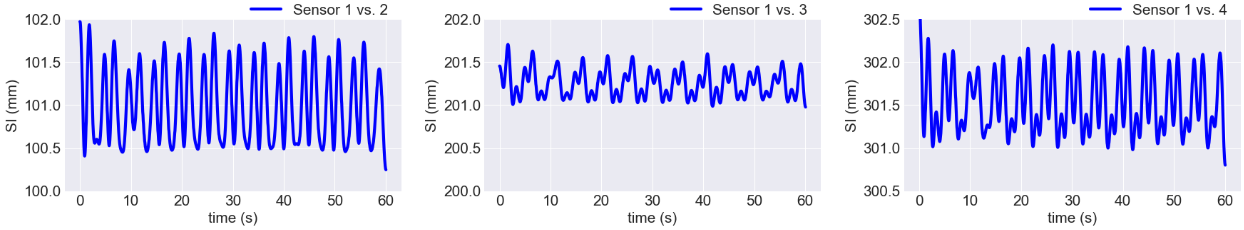

Table 7A: Sensors' distances comparison when gantry angle = 180 degrees

Metrics (mm)	Sensor 1 vs. 2	Sensor 1 vs. 3	Sensor 1 vs. 4
**mean (mm)**	101.146127	201.410292	301.608228
**std (mm)**	0.435283	0.148526	0.362587

Figure 7A. Sensors Distances for Moving Plateform at Gantry Angle 180^{0}

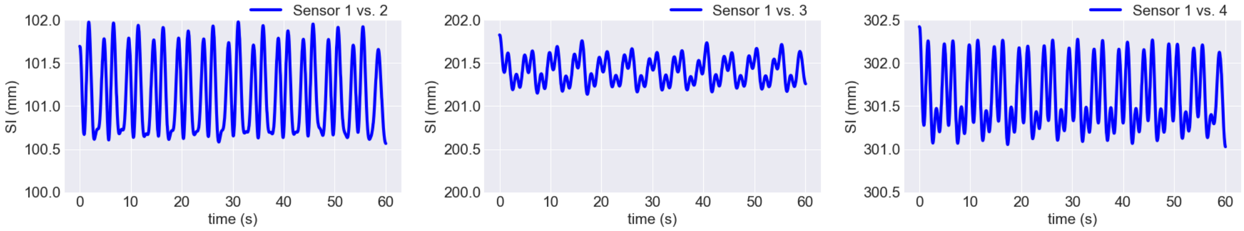

Table 8A: Sensors' distances comparison when gantry angle = 270 degrees

Metrics (mm)	Sensor 1 vs. 2	Sensor 1 vs. 3	Sensor 1 vs. 4
**mean (mm)**	101.180776	201.501215	301.758232
**std (mm)**	0.429507	0.142858	0.341013

Figure 8A. Sensors Distances for Moving Plateform at Gantry Angle 270^{0}

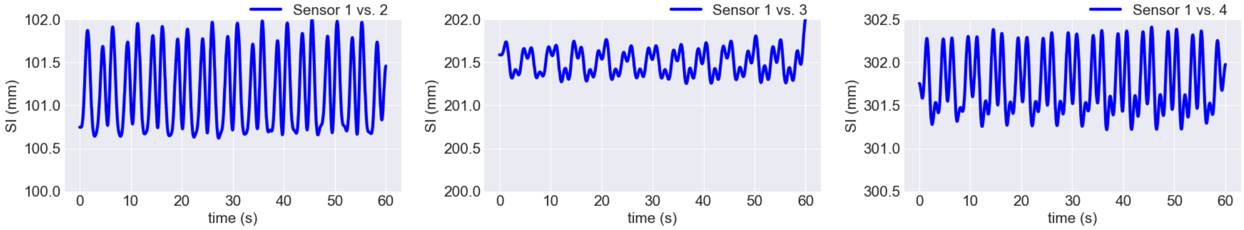

Condition3: Moving Gantry and Static Motion Platform

Table 9A: Sensors' distances comparison when gantry rotates clockwise

Metrics (mm)	Sensor 1 vs. 2	Sensor 1 vs. 3	Sensor 1 vs. 4
**mean (mm)**	100.556080	201.240761	301.256441
**std (mm)**	0.083696	0.107756	0.067903

Figure 9A. Sensors Distances for Static Platform Clockwise Gantry Rotation

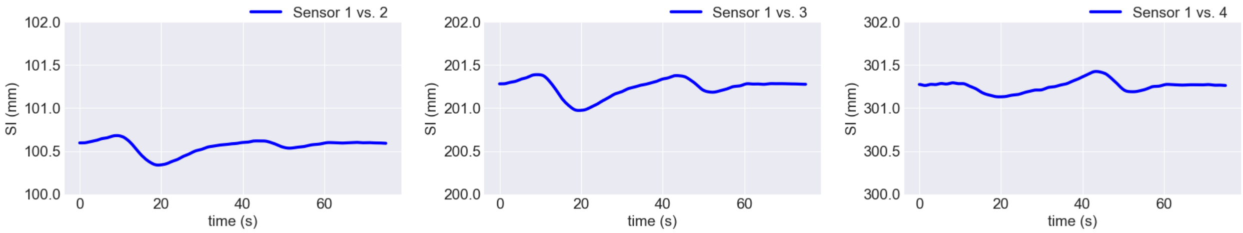

Table 10A: Sensors' distances comparison when gantry rotates anti-clockwise

Metrics (mm)	Sensor 1 vs. 2	Sensor 1 vs. 3	Sensor 1 vs. 4
**mean (mm)**	100.556626	201.241554	301.257674
**std (mm)**	0.082832	0.106605	0.068248

Figure 10A. Sensors Distances for Static Platform Anti-Clockwise Gantry Rotation

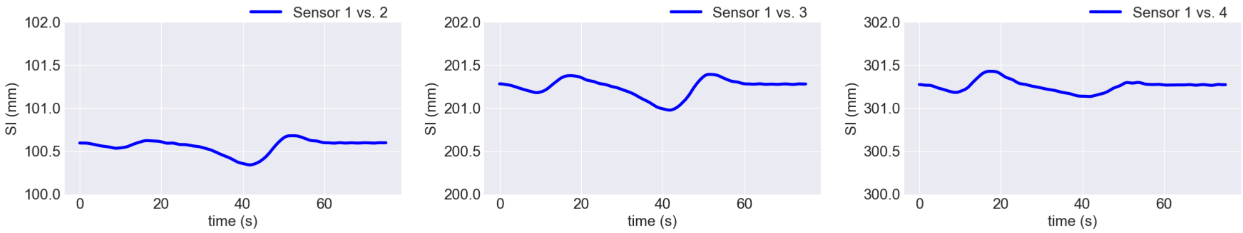

Condition4: Moving Gantry and Moving Motion Platform

Table 11A: Sensors' distances comparison when gantry rotates clockwise

Metrics (mm)	Sensor 1 vs. 2	Sensor 1 vs. 3	Sensor 1 vs. 4
**mean (mm)**	101.120436	201.371532	301.598075
**std (mm)**	0.441939	0.181752	0.370767

Figure 11A. Sensors Distances for Moving Platform Clockwise Gantry Rotation

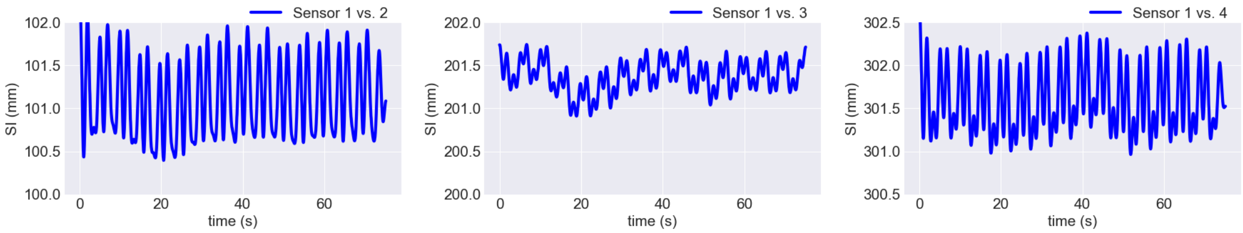

Table 12A: Sensors' distances comparison when gantry rotates anti-clockwise

Metrics (mm)	Sensor 1 vs. 2	Sensor 1 vs. 3	Sensor 1 vs. 4
**mean (mm)**	101.122381	201.373496	301.602828
**std (mm)**	0.431847	0.185111	0.368656

Figure 12A. Sensors Distances for Moving Platform Anti-Clockwise Gantry Rotation

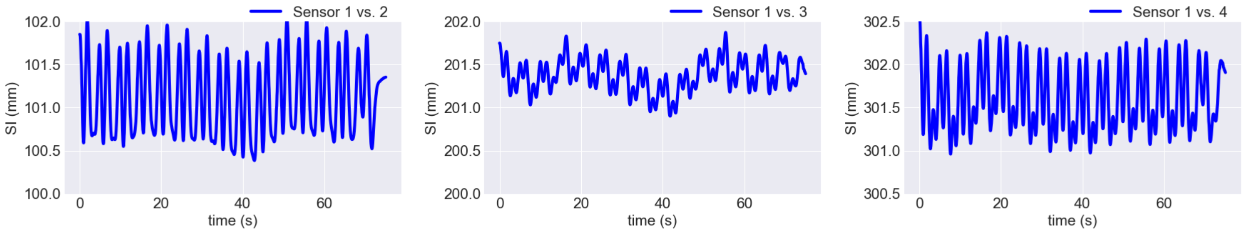

Condition5: Analyzing the Effect of Radiation

Table 13A: Sensors' distances comparison when radiation beam is ON

Metrics (mm)	Sensor 1 vs. 2	Sensor 1 vs. 3
**mean (mm)**	100.456523	200.184736
**std (mm)**	0.225127	0.336652

Figure 13A. Sensors Distances for Analyzing the Effect of Radiation Beam

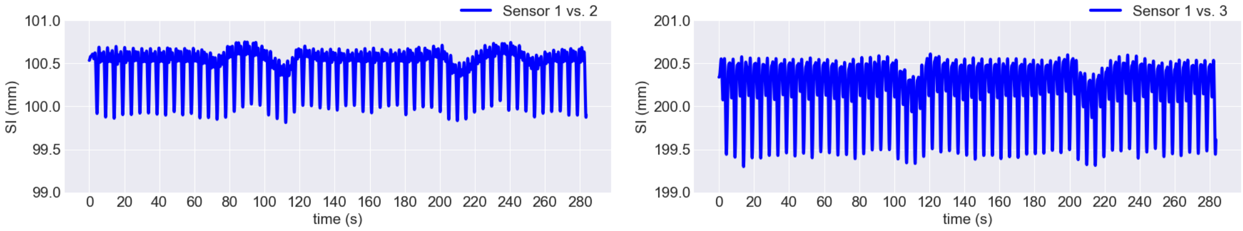

Condition6: Analyzing the Effect of CBCT Acquisition

Table 14A: Sensors' distances comparison for CBCT Acquisition

Metrics (mm)	Sensor 1 vs. 2	Sensor 1 vs. 3
**mean (mm)**	101.180542	201.986503
**std (mm)**	0.087929	0.223267

Figure 14A. Sensors Distances for CBCT Acquisition

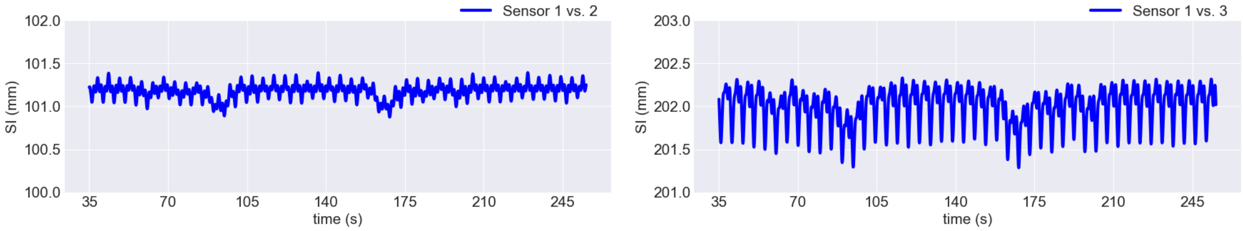

Supplement: Supplementary file 1 — Supporting Information [file ACM2-26-e70187-s001.pdf]
